# A Randomized, Double‐Blind, 2‐Treatment, 2‐Period, Crossover Phase 1 Study to Compare the Pharmacokinetics, Safety and Tolerability of 60 IU/Kg of Abcertin and Cerezyme in Healthy Volunteers Following a Single Intravenous Administration

**DOI:** 10.1002/mgg3.70111

**Published:** 2025-06-13

**Authors:** Eungu Kang, Dohyung Kim, Soojin Hwang, Charlotte Lemech, Jessica Wharton, Yongyoon Lee, Han Wook Yoo, Beom Hee Lee

**Affiliations:** ^1^ Department of Pediatrics Korea University Ansan Hospital, Korea University College of Medicine Ansan Republic of Korea; ^2^ Department of Pediatrics Asan Medical Center Children's Hospital, University of Ulsan College of Medicine Seoul Republic of Korea; ^3^ Scientia Clinical Research (SCR) Randwick New South Wales Australia; ^4^ ISU ABXIS Co., Ltd. Seongnam‐si Gyeonggi‐do Republic of Korea; ^5^ Department of Pediatrics CHA University School of Medicine, Bundang CHA Medical Center Seongnam Republic of Korea; ^6^ Medical Genetics Asan Medical Center, University of Ulsan College of Medicine Seoul Republic of Korea

**Keywords:** biosimilar, enzyme replacement therapy, Gaucher disease, pharmacokinetics, safety

## Abstract

**Background:**

Imiglucerase (Cerezyme; Sanofi, Paris, France), an analogue of β‐glucocerebrosidase produced by recombinant DNA technology, has been a safe and effective treatment for Gaucher disease (GD) for over 25 years. A new imiglucerase, Abcertin (Seongnam‐si, Gyeonggi‐do, Republic of Korea) has shown a similar safety and efficacy profile in previous clinical studies. This study compared the pharmacokinetics, immunogenicity, safety, and tolerability to EU‐sourced Cerezyme following a single 60 IU/kg dose.

**Methods:**

This phase 1, single‐center, randomized, double‐blind, two‐way crossover study enrolled 36 healthy volunteers aged 18–45 years. Participants were randomly assigned to receive either Abcertin or Cerezyme in a predetermined sequence.

**Results:**

Abcertin reached peak plasma concentrations at a median *t*_max_ of 61 min (range: 40–121 min). The mean *C*_max_, AUC_0–last_, and AUC_0–inf_ were 115.4 mU/mL, 12,190 min·mU/mL, and 12,210 min mU/mL, respectively, indicating bioequivalence to Cerezyme. The mean *t*_½_, CL, and *V*_z_ were 6.88 min, 376.7 mL/min, and 3.62 L, respectively, and were comparable between the two treatments. One participant in the Cerezyme group developed anti‐drug antibodies, which were non‐neutralizing A total of 24 subjects experienced treatment‐emergent adverse event (TEAE). The most common TEAE was headache (3 in the Abcertin group and 5 in the Cerezyme group), followed by general disorders and administration site condition (3 in Abcertin group and 5 in Cerezyme group). Two participants in the Cerezyme sequence experienced severe TEAEs: one had a urinary tract infection, and the other developed urticaria, which leading to study withdrawal.

**Conclusion:**

Abcertin demonstrated pharmacokinetic equivalence to Cerezyme, with a comparable safety, immunogenicity, and tolerability profile. These findings support its potential as an affordable biosimilar for GD treatment.

## Graphical abstract

This study evaluates the pharmacokinetic equivalence, immunogenicity, and safety profiles of Abcertin and Cerezyme in healthy volunteers. It demonstrated the pharmacokinetic bioequivalence of Abcertin to Cerezyme, highlighted favorable safety and tolerability profiles with no clinically significant adverse events for Abcertin, and confirmed its low immunogenic potential.

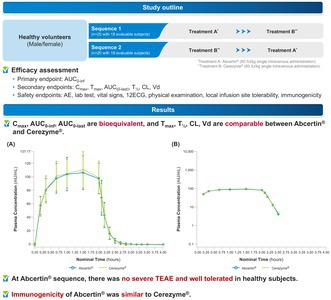

## 1 Introduction

Gaucher disease (GD), an autosomal recessive disorder caused by a deficiency in the lysosomal enzyme glucocerebrosidase (GBA) encoded by *GBA1* gene, is the most common glycolipid storage disorder, with an overall incidence of approximately 1 in 40,000 to 60,000 (Grabowski et al. 2004; Stirnemann et al. 2017). The incidence is significantly higher in individuals of Ashkenazi Jewish ancestry, affecting approximately 1 in 850 births due to a higher carrier frequency of the disease‐causing gene (Horowitz et al. 1998). The accumulation of lipid glucocerebroside within the lysosomal compartment of macrophages causes functional impairment of internal organs, including the bone marrow, spleen, liver, and nervous system. The clinical phenotypes of GD are categorized into three forms, although the full disease spectrum is a continuum. Type 1 is characterized by the absence of neurological symptoms, whereas types 2 and 3 are characterized by neurological involvement (Charrow et al. 2000; Mistry et al. 2011; Tajima et al. 2009). The clinical presentation of type 1 GD varies widely, ranging from asymptomatic to severely debilitating (Biegstraaten et al. 2008; Tajima et al. 2009). Most symptomatic patients exhibit visceral, hematologic, and skeletal manifestations, including anemia, thrombocytopenia, hepatosplenomegaly, bone pain, and delayed growth and puberty. Type 3 GD, also known as subacute or chronic neuronopathic GD, presents with moderate systemic involvement, along with supranuclear saccadic horizontal gaze palsy, progressive myoclonus epilepsy, cerebellar ataxia, or spasticity (Daykin et al. 2021; Hwang et al. 2024; Kim et al. 2020; Roshan Lal and Sidransky 2017). Type 2 GD, the acute neuronopathic form, is characterized by early‐onset and severe neurological deterioration, accompanied by systemic hepatosplenomegaly.

The long‐term safety and effectiveness of enzyme replacement therapy (ERT) in GD have been well established, particularly in improving bone, hematologic, and visceral disease parameters (Nabizadeh et al. 2018; Shemesh et al. 2015). However, rare manifestations of GD, such as mesenteric lymphadenopathy, Gaucheroma, interstitial pneumonitis, and neurological manifestations are not responsive to ERT (Kim et al. 2022; Ramaswami et al. 2021). Following the approval of an enzyme extracted from human placenta (alglucerase; Ceredase, Genzyme Corp., Cambridge, MA, USA) by the Food and Drug Administration (FDA) in 1991 for the treatment of type 1 GD, Imiglucerase (Cerezyme, Genzyme Corp), a recombinant DNA‐produced analog of human GBA using Chinese Hamster Ovary (CHO) cells that can be produced in large quantities, was approved in 1994 (Barton et al. 1991; Zimran et al. 1995). Currently, two other recombinant enzymes have been developed: velaglucerase (Vpriv, Shire) produced using human fibroblasts and taliglucerase (Elelyso, Pfizer) produced using carrot cells (Zimran et al. 2010, 2011). A newly developed form of imiglucerase, Abcertin (ISU Abxis, Seongnam‐si, Gyeonggi‐do, Republic of Korea), has been characterized in vivo and in vitro for its structural, physicochemical, immunological, and biological properties. Previous clinical studies have demonstrated its safety and efficacy profiles to be comparable to those of Cerezyme (Choi et al. 2015; Lee et al. 2017). However, for Abcertin to be approved as a biosimilar under World Health Organization (WHO) or European Medicines Agency (EMA) guidelines, comparative pharmacokinetic (PK) studies with the reference product in healthy volunteers are required. To meet this regulatory requirement, the present study evaluated the PK, immunogenicity, short‐term safety, and tolerability of Abcertin compared to Cerezyme following a single‐dose (60 IU/kg) administration in healthy volunteers.

## 2 Methods

### 2.1 Ethical Compliance

This study was conducted in accordance with the ethical principles of the Declaration of Helsinki and the Guidelines of the International Council for Harmonization Guideline for Good Clinical Practice, and all applicable laws or regulations. Th study protocol was approved by the Bellberry Human Research Ethics Committee, and written informed consent was obtained from all participants before any study procedures were performed.

### 2.2 Study Design

This was a phase 1, single‐center, randomized, double‐blind, two‐way crossover study evaluating Abcertin and Cerezyme in healthy volunteers aged 18–45 years. In line with bioequivalence guidelines recommending studies using the highest strength, a single intravenous infusion of imiglucerase at 60 IU/kg—the highest approved initial dose for GD—was selected (Agency 2010). Participants were randomly assigned to receive either Treatment A (Abcertin, 60 IU/kg intravenously) followed by Treatment B (Cerezyme, 60 IU/kg intravenously) or vice versa, with a crossover design.

**FIGURE 1:**
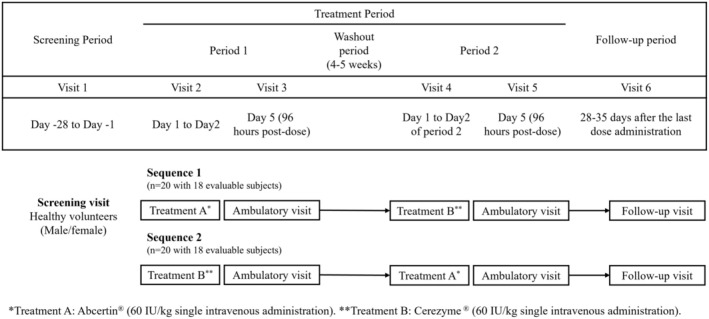
Overview of the study structure.

The study included a total of 6 visits; two of which were inpatient visits (Visit 2 and Visit 4; 24 h post‐dose). The 2 treatment periods were separated by a washout of at least 28 days but not more than 35 days, a duration chosen to fully minimize potential pharmacodynamic and immunological carryover effects resulting from macrophage uptake, despite the short plasma half‐life of imiglucerase. Follow‐up visits occurred 28–35 days after administration during Treatment Period 2 (Figure 1). The two products were administered as a single intravenous infusion of 60 IU/kg over 2 h at an infusion rate of 0.5 IU/kg/min. The start and end times of the intravenous infusions were documented in the case report forms, along with details of any interruptions (stop time/restart time), the volume infused, and any adjustments in the infusion rate. In case of infusion interruption, a PK blood sample was collected prior to restarting the infusion, followed by continuation of the designated time collection for the rest of the infusion. Infusions were administered on alternate arms of the participant across the 2 treatment periods.

### 2.3 Study Participants

The study population consisted of healthy male and female volunteers who met all the inclusion criteria and none of the exclusion criteria. Participants were aged 18–45 years, with a body mass index of 18.5–30 kg/m^2^ and a weight of 55–105 kg, as determined by the Principal Investigator based on medical history, physical examination, 12‐lead electrocardiography (ECG), and laboratory evaluations (including hematology, blood chemistry, coagulation, and urinalysis tests). Participants were excluded if they were taking any investigational drugs in another study, or who were on extended follow‐up, had ongoing symptoms of acute diseases within 28 days prior to Period 1, or had any medical history that could have affected the drug distribution, metabolism, and excretion. Additional inclusion and exclusion criteria are provided in Table S1.

### 2.4 Pharmacokinetic and Safety Measurements

The PK analysis set included all randomized participants who received both Abcertin and Cerezyme according to protocol, had at least one quantifiable post‐dose concentration, and had no major protocol deviations or events that could significantly affect PK analysis. Participants who tested positive for anti‐drug antibodies (ADAs) during the pre‐dose assessment of Treatment Period 1 were excluded. The schedule of assessments is shown in Table S2. The time points of assessment for PK studies of Abcertin and Cerezyme were within 30 min prior to infusion, and at 10, 20, 40, 60, 90, 120 min during infusion, and at 5, 10, 20, 30, 40, 50, 60, 70, 80, 90, and 120 min after the end of infusion. The primary PK endpoint was the area under the concentration–time curve (AUC) from time zero to infinity (AUC_0–inf_). Secondary PK endpoints included maximum plasma concentration (*C*_max_), time to *C*_max_ (*t*_max_), AUC from time zero to the last measurable plasma concentration (AUC_0–last_), terminal half‐life (*t*_½_), total body clearance (CL), and volume of distribution in the terminal phase (*V*_z_).

Participants who received at least one dose of Abcertin or Cerezyme were included in the safety analysis. Safety evaluations were performed at pre‐ and post‐dosing, and at every visit. These evaluations included a physical examination, measurements of vital signs, 12‐lead ECG, clinical laboratory tests, injection site reactions and adverse events (AEs). AEs were coded according to the Medical Dictionary for Regulatory Activities (MedDRA; version 22.1). For immunogenicity assessments, ADA levels were measured before dosing and at the follow‐up visit. The ADA analysis set included all randomized participants who received both Abcertin and Cerezyme per protocol and had available ADA data. Participants who received treatment in only one period and subsequently withdrew were excluded. Samples that tested positive in the screening assay were subjected to a confirmatory assay. Those confirmed positive were further evaluated for antibody titer and neutralizing capacity. PK and neutralizing antibodies(NAbs) analyses were conducted using fluorescent enzyme activity assay platforms, ADA analyses were performed using an electrochemiluminescence (ECL)‐based assay format.

### 2.5 Statistical Analysis

All analyses were performed using SAS version 9.3 or later in a secure and validated environment. PK analyses were conducted using Phoenix WinNonLin (WNL) version 8.0 or later (Certara USA Inc., Princeton, NJ). Descriptive statistics for the primary endpoint (AUC_0–inf_) were presented by treatment and period, including the number of participants (*N*), mean, standard deviation (SD), percent coefficient of variation (%CV), median, minimum and maximum values, geometric mean, and geometric %CV. PK biosimilarity between Abcertin and Cerezyme was assessed using an analysis of variance (ANOVA) on log‐transformed AUC_0–inf_ values. The ANOVA model included fixed effects for treatment, period, sequence, and participants nested within the sequence. The point estimates and 90% confidence intervals (CIs) for the difference among treatments were constructed. The point estimates and 90% CIs were then exponentially back‐transformed to provide point and 90% CI estimates for the ratio (for logarithmically‐transformed data) of geometric least squares (LS) means for imiglucerase AUC_0–inf_ for Abcertin versus Cerezyme. PK biosimilarity of Abcertin was concluded relative to each source of Cerezyme if the 90% CI fell entirely within 80.00% to 125.00% for AUC_0–inf_. As a sensitivity analysis, an additional analysis was performed using subject within sequence as a random effect instead of as a fixed effect. Descriptive statistics for each secondary PK endpoint were summarized by treatment and period, and the same ANOVA model was applied to *C*_max_ and AUC_0–last_. A sensitivity analysis using a random effect for participants nested within the sequence was also conducted.

## 3 Results

### 3.1 Baseline Characteristics

Of the 84 participants screened, 42 were randomized, with 38 completing the study; 2 discontinued due to consent withdrawal, 1 due to ineligibility, and 1 due to severe urticaria. The numbers of participants in the safety, PK, and ADA populations were 42, 37, and 38, respectively. Demographic and baseline characteristics are summarized in Table 1.

**TABLE 1:** The demographic and baseline characteristics of participants.

	Total (*N* = 42)
Age (years)	26.6 ± 7.49
Sex (Male, *N*, %)	29 (69.05)
Race (*N*, %)	
White	32 (76.19)
Australian Native	0 (0)
Asian	8 (19.05)
Other Pacific Islander	0 (0)
Black	0 (0)
Other	2 (4.76)
Height (cm)	173.96 ± 9.64
Weight (kg)	72.42 ± 12.04
BMI (kg/m^2^)	23.86 ± 2.87

Abbreviation: BMI, body mass index.

### 3.2 Pharmacokinetic Evaluations

**FIGURE 2:**
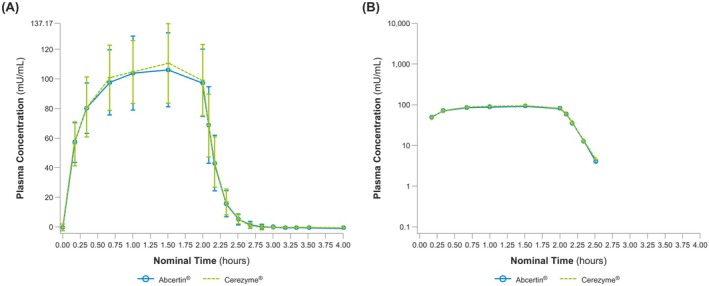
Pharmacokinetic Results. (A) Arithmetic mean ± SD (linear scale) and (B) geometric mean (semi‐logarithmic scale).

One participant experienced an interrupted infusion and an unscheduled sampling time point during Treatment Period 2, resulting in the exclusion of their PK data from the summary statistics. As a result, 37 participants were included in the PK analysis set. The mean concentrations of Abcertin and Cerezyme over 4 h post‐dose are shown in Figure 2.

Overall, PK parameters were comparable between Abcertin and Cerezyme (Table 2). For Abcertin, peak plasma concentrations were reached at a median *t*_max_ of 61.0 min (range, 40–120 min). The mean values for *C*_max_, AUC_0–inf_, and AUC_0–last_ of Abcertin were 115.4 ± 23.8 mU/mL, 12,210 ± 2483 min·mU/mL, and 12,190 ± 2480 min·mU/mL, respectively. The mean values for *t*_1/2_, CL, and *V*_z_ were 6.88 ± 1.14 min, 363.7 ± 124.3 mL/min, and 3.62 ± 0.87 L, respectively. Inter‐subjective variability for *C*_max_, AUC_0–inf_, and AUC_0–last_ were 20.6%, 20.3%, and 20.3%, respectively. For Cerezyme, the median *t*_max_ was 60 min (range, 20–120 min). The mean values for *C*_max_, AUC_0–inf_, and AUC_0–last_ of Cerezyme were 117.5 ± 24.7 mU/mL, 12,440 ± 2514 min·mU/mL, and 12,320 ± 2513 min·mU/mL, respectively. The mean values for *t*_1_/_2_, CL, and *V*_z_ were 7.20 ± 0.91 min, 368.2 ± 116.0 mL/min, and 3.77 ± 1.04 L, respectively. Inter‐subject variabilities for *C*_max_, AUC_0–inf_, and AUC_0–last_ were 21.1%, 20.2%, and 20.2%, respectively.

**TABLE 2:** Plasma pharmacokinetic parameters.

	Abcertin							Cerezyme						
	Mean ± SD	CV(%)^a^	Median	Min	Max	Geo mean	Geo CV (%)^b^	Mean ± SD	CV(%)^a^	Median	Min	Max	Geo mean	Geo CV (%)^b^
*C*_max_ (mU/mL)	115.4 ± 23.8	20.6	115.0	68.1	172.0	112.8	22.1	117.5 ± 24.7	21.1	118.0	72.5	184.0	115.0	21.1
*t*_max_ (min)			61.0	40.0	121.0					60.0				
AUC_0–inf_ (min·mU/mL)	12,210 ± 2483	20.3	12480.0	7300.0	19000.0	11960.0	21.5	12,440 ± 2514	20.2	12260.0	7480.0	19200.0	12190.0	20.9
AUC_0–last_ (min·mU/mL)	12,190 ± 2480	20.3	12470.0	7290.0	189000.0	11940.0	21.5	12,320 ± 2513	20.2	12230.0	7460.0	19200.0	12170.0	20.9
*t*_1/2_ (min)	6.882 ± 1.139	16.6	6.8	5.1	10.0	6.797	15.9	7.204 ± 0.9135	12.7	7.3	5.5	9.4	7.147	12.9
CL (mL/min)	363.7 ± 124.3	33	323.7	235.0	705.0	360.0	30.2	368.2 ± 116.0	31.5	317.7	203.0	715.0	353.2	28.9
*V*_z_ (L)	3.624 ± 0.8695	24	3.2	2.4	5.7	3.5	23.2	3.770 ± 1.039	27.6	3.4	2.2	7.1	3.6	26.8

Abbreviations: AUC_0–inf_, area under the concentration‐time curve from pre‐dose to infinite time; AUC_0–last_, area under the plasma drug concentration‐time curve from time zero to last measurable concentration; CL, total body clearance; C_max_, maximum plasma concentration; CV, coefficient of variation; Geo CV, geometric coefficient of variation; Geo Mean, geometric mean; *t*_½_, terminal half‐life; *t*_max_, time to achieve *C*_max_; *V*_z_, volume of distribution.

^a^ CV (%) = (SD/Mean) × 100.

^b^ Geo CV (%) = sqrt(exp(v)—1) × 100, where v is the variance of ln(concentration).

Abcertin (60 IU/kg) was bioequivalent to Cerezyme (60 IU/kg) with respect to AUC_0–inf_, AUC_0–last_, and *C*_max_, as the corresponding 90% CIs for geometric mean ratios (GMRs) were entirely within the predefined bioequivalence range of 80%–125% (Table 3).

**TABLE 3:** Bioequivalence between Abcertin and Cerezyme.

Parameters	Geometric least square mean		Geometric mean ratio (%) (Abcertin/Cerezyme) × 100	90% CI
	Abcertin	Cerezyme		
AUC_0–inf_ (min*mU/mL)	11,950	12,170	98.18	94.57–101.92
AUC_0–last_ (min*mU/mL)	11,930	12,160	98.15	94.55–101.90
*C*_max_ (mU/mL)	112.7	115	98.05	93.86–102.42

In a sensitivity analysis, the test/reference GMRs for AUC_0–inf_, AUC_0–last_, and *C*_max_ were 98.18%, 98.15%, and 98.05%, respectively, with their corresponding 90% CIs fully contained within the bioequivalence range of 80%–125% (94.57%–101.92% for AUC_0–inf_, 94.55%–101.90% for AUC_0–last_, and 93.86%–102.42% for *C*_max_).

One participant (number 10118) experienced an interrupted infusion but restarted infusion approximately 10 min during Treatment Period 2. A sensitivity analysis excluding this participant's data to assess its impact on the overall PK analysis yielded the same bioequivalence conclusion, as the bioequivalence criteria for AUC_0–inf_, AUC_0–last_, and *C*_max_ remained satisfied. The test/reference GMRs were 98.37%, 98.34%, and 98.12%, respectively, with 90% CIs fully within the bioequivalence range of 80%–125% (94.67%–102.21% for AUC_0–inf_, 94.64%–102.19% for AUC_0–last_, and 93.81%–102.63% for *C*_max_).

### 3.3 Immunogenicity Evaluation

In immunogenicity evaluation, all 19 participants who received Abcertin in Sequence 1 were negative for ADA presence at Day 1 pre‐dose in Treatment Period 2. On the other hand, 1 out of 19 participants who received Cerezyme in Sequence 1 were positive for treatment‐induced ADA at Day 1 pre‐dose in Treatment Period 2, which was not positive in NAbs. All 35 follow‐up immunogenicity samples were negative for ADA.

### 3.4 Safety and Tolerability

The safety analysis set included all participants (*N* = 42) who received at least one dose of Abcertin or Cerezyme. All reported AEs were mild or moderate in severity, and no serious adverse event occurred during the study. Overall, 24 (57.14%) treatment‐emergent adverse events (TEAEs) were reported: 18 (42.86%) in the Abcertin sequence and 18 (42.86%) in the Cerezyme sequence. Table 4 summarizes the TEAEs related to the treatment. Fifteen participants (14 in the Abcertin group and 5 in the Cerezyme group) experienced mild TEAEs, while seven participants (4 in the Abcertin group and 3 in the Cerezyme group) experienced moderate TEAEs. Two severe TEAEs were in the Cerezyme sequence: one participant developed a severe urinary tract infection, and another experienced severe urticaria, leading to study withdrawal. No AEs resulted in death. No clinically significant changes were observed in vital signs, laboratory values, or ECG findings, and no signs of hepatotoxicity were detected.

**TABLE 4:** Summary of treatment‐emergent adverse events (TEAEs) related to the investigational product.

	Abcertin	Cerezyme	Total (*N* = 42)
Participants with ≥ 1 TEAE	5 (11.9)	9 (21.4)	13 (31.0)
Nervous system disorders	2 (4.8)	5 (11.9)	7 (16.7)
Headache	2 (100)	5 (100)	7 (100)
Gastrointestinal disorders	2 (4.8)	2 (4.8)	3 (7.1)
Dyspepsia	1 (50.0)	1 (50.0)	2 (66.7)
Vomiting	1 (50.0)	1 (50.0)	1 (33.3)
Diarrhea	0	1 (50.0)	1 (33.3)
Skin and subcutaneous tissue disorders	1 (2.4)	1 (2.4)	2 (4.8)
Rash	1 (100)	0	1 (50.0)
Urticaria	0	1 (100)	1 (50.0)
Blood and lymphatic system disorders	0	1 (2.4)	1 (2.4)
Lymphopenia	0	1 (100)	1 (100)
General disorders and administration site conditions	0	1 (2.4)	1 (2.4)
Injection site erythema	0	1 (100)	1 (100)

## 4 Discussion

Abcertin was approved in Iran, Peru, Bolivia, Colombia, Ecuador, Venezuela, Algeria and South Korea for the treatment of GD through a national regulatory pathway that permitted the submission of Phase 3 data post‐approval. The clinical efficacy, PK, and safety data for Abcertin were reported in published Phase 2 and Phase 3 studies. However, according to WHO and EMA guidelines, comparative PK studies in healthy volunteers are mandatory for biosimilar approval.

This study evaluated the PK equivalence of Abcertin and Cerezyme in healthy adults. Following intravenous administration of a single 60 IU/kg dose, the PK parameters demonstrated bioequivalence between Abcertin and Cerezyme, based on the primary PK endpoint, AUC_0–inf_, with an Abcertin/Cerezyme GMR of 98.18% and 90% CIs within the predefined bioequivalence range of 80%–125% (94.57%–101.92% for AUC_0–inf_). Additional PK parameters, AUC_0–last_ and *C*_max_, also met bioequivalence criteria using the same ANOVA model, with 90% CIs similarly contained within the predefined range. A sensitivity analysis, using an ANOVA model with subject within sequence as a random effect, confirmed these results. Other PK parameters, including *t*_max_, *t*_½_, CL, and *V*_z_, were also similar between Abcertin and Cerezyme.

Improvements in hematological, visceral, and bone parameters, as well as quality of life, have been well established following imiglucerase therapy in patients with GD (Serratrice et al. 2016; Weinreb et al. 2013; Zimran et al. 2018). The efficacy of Abcertin in patients with type 1 GD was comparable to that of Cerezyme in a Phase 2, multicenter, open‐label, switch‐over trial (Choi et al. 2015). Additionally, a multicenter, open‐label Phase 3 study demonstrated the efficacy of Abcertin by significantly increasing hemoglobin concentration and platelet count while decreasing organ volume, acid phosphatase, and chemokine ligand 18 levels (Lee et al. 2017). Also, long‐term combination therapy with Abcertin 60 IU/kg and high‐dose ambroxol (35 mg/kg/day) has shown promising results in type 3 GD (Hwang et al. 2024; Kim et al. 2020).

In a phase 2 study, the PK profile of Abcertin after intravenous administration of 30–60 U/kg over 90 min demonstrated a *T*_max_ of 1.34 ± 0.32 h (range, 1.00–1.67 h) and a clearance half‐life of 0.11 ± 0.01 h (range, 0.09–0.12 h) (Choi et al. 2015). The elimination half‐life, plasma clearance, and volume of distribution of imiglucerase are independent of infusion time and dose within a range of 7.5–60 U/kg (Edmunds 2005). The plasma activity half‐life is short, ranging from 3.6 to 10.4 min, which is advantageous due to the rapid inactivation of glucocerebrosidase at the neutral pH of blood and the stabilization of enzymatic activity for lysosomal trafficking via rapid endocytosis (Weinreb 2008). The uptake and tissue distribution of iodine‐123‐labeled imiglucerase or aglucerase in eight patients with type 1 GD and one healthy control showed rapid blood clearance with a half‐life of 4–7 min and significant uptake in the liver (30%), spleen (15%), and bone marrow. Bone marrow clearance had a half‐life of 14.1 h, whereas visceral clearance followed a biphasic pattern, with half‐lives of 1–2 h and 34–42 h (Mistry et al. 1996).

The long‐term safety and immunogenicity of imiglucerase have been reported with no serious adverse events reported in clinical trials or in the International Cooperative Gaucher Group (ICCG) Gaucher registry (Starzyk et al. 2007; Weinreb 2008). Good tolerance to imiglucerase has also been confirmed in switch and non‐inferiority studies comparing other ERTs, including velaglucerase and taliglucerase (Elstein et al. 2015; Pastores et al. 2016; Smith et al. 2016). Overall, ERT is considered safe for all ages and GD subtypes, with no reported death or irreversible damage related to treatment (Gupta and Pastores 2018; Starzyk et al. 2007; Zimran et al. 1995). A few cases of anaphylactic reactions have been reported, typically during the first or second infusion, and were efficiently managed by premedication, slowing the infusion rate, or switching to another ERT. Most AEs, including chills, fever, pruritus, rashes, urticaria, and dyspnea, were mild to moderate in severity, transient, and did not lead to discontinuation of therapy. In this study, 24 participants (57.14%) experienced at least one TEAE. Headache was the most frequently reported TEAE considered treatment‐related by the investigator. All adverse events observed with Abcertin treatment were mild or moderate in severity, with no clinically relevant abnormalities observed in laboratory tests, vital signs, or ECG. Two severe TEAEs, considered by the investigator to be treatment‐related, were reported in the Cerezyme treatment sequence. Notably, one participant experienced urticaria, a special‐interest adverse event, leading to study withdrawal. The incidence of treatment‐induced ADAs was low following a single intravenous administration of Abcertin or Cerezyme; only 1 participant after receiving Cerezyme in sequence 2 was positive in ADA without neutralizing capacity. In a comparative trial, ADAs developed in 6 of 15 patients receiving alglucerase and in 3 of 15 patients receiving imiglucerase. While antibodies appeared within 3–6 months in the imiglucerase group, no major immunological effects or diminished therapeutic responses were observed in either group (Grabowski et al. 1995).

## 5 Conclusion

Cerezyme, an ERT for GD, has been shown to be effective and safe for disease management. However, its high cost significantly limits access, particularly in resource‐limited settings. Introducing biosimilars could improve treatment affordability and availability, thereby expanding access to effective therapies. By meeting key biosimilarity criteria, Abcertin may offer a more cost‐effective alternative, increasing accessibility for patients with GD.

Following the administration of a single dose to healthy adults, Abcertin demonstrated PK equivalence to Cerezyme based on the study's primary endpoints. Secondary PK parameters, immunogenicity, and safety profiles were also comparable between the two groups. These findings provide evidence that Abcertin could serve as a potential biosimilar to Cerezyme.

## Author Contributions

C.L., J.W., Y.L., H.W.Y., B.H.L. contributed to the study design. C.L., J.W., Y.L. contributed to data collection. E.K., C.L., J.W., Y.L., and B.H.L. contributed to data analysis or interpretation. E.K. and B.H.L. drafted the manuscript. All authors critically reviewed and revised the manuscript, approved the final manuscript for publication.

## Ethics Statement

The study was conducted in accordance with the ethical principles of the Declaration of Helsinki and the Guidelines of the International Council for Harmonization Guideline for Good Clinical Practice, and all applicable laws or regulations.

## Consent

This study was approved by the Bellberry Human Research Ethics Committee and written informed consent was obtained from all participants before any study procedures.

## Conflicts of Interest

Y.L. is an employee of ISU ABXIS. E.K., D.K., S.H., C.L., J.W., H.W.Y., and B.H.L. declare no conflicts of interest.

## Supporting information


**Table S1.** Selection criteria for the study population.
**Table S2.** Schedule of Assessments.

## Data Availability

Anonymized data not included in this article will be shared by request from any qualified investigator.

## References

[mgg370111-bib-0002] Barton, N. W. , R. O. Brady , J. M. Dambrosia , et al. 1991. “Replacement Therapy for Inherited Enzyme Deficiency—Macrophage‐Targeted Glucocerebrosidase for Gaucher's Disease.” New England Journal of Medicine 324, no. 21: 1464–1470. 10.1056/nejm199105233242104. 2023606

[mgg370111-bib-0003] Biegstraaten, M. , I. N. van Schaik , J. M. Aerts , and C. E. Hollak . 2008. “Non‐Neuronopathic Gaucher Disease Reconsidered. Prevalence of Neurological Manifestations in a Dutch Cohort of Type I Gaucher Disease Patients and a Systematic Review of the Literature.” Journal of Inherited Metabolic Disease 31, no. 3: 337–349. 10.1007/s10545-008-0832-y. 18404411

[mgg370111-bib-0004] Charrow, J. , H. C. Andersson , P. Kaplan , et al. 2000. “The Gaucher Registry: Demographics and Disease Characteristics of 1698 Patients With Gaucher Disease.” Archives of Internal Medicine 160, no. 18: 2835–2843. 10.1001/archinte.160.18.2835. 11025794

[mgg370111-bib-0005] Choi, J. H. , B. H. Lee , J. M. Ko , et al. 2015. “A Phase 2 Multi‐Center, Open‐Label, Switch‐Over Trial to Evaluate the Safety and Efficacy of Abcertin in Patients With Type 1 Gaucher Disease.” Journal of Korean Medical Science 30, no. 4: 378–384. 10.3346/jkms.2015.30.4.378. 25829804 PMC4366957

[mgg370111-bib-0006] Daykin, E. C. , E. Ryan , and E. Sidransky . 2021. “Diagnosing Neuronopathic Gaucher Disease: New Considerations and Challenges in Assigning Gaucher Phenotypes.” Molecular Genetics and Metabolism 132, no. 2: 49–58. 10.1016/j.ymgme.2021.01.002. 33483255 PMC7884077

[mgg370111-bib-0007] Edmunds, T. 2005. “β‐Glucocerebrosidase Ceredase and Cerezyme.” In Directory of Therapeutic Enzymes, 117–134. CRC press.

[mgg370111-bib-0008] Elstein, D. , A. Mehta , D. A. Hughes , et al. 2015. “Safety and Efficacy Results of Switch From Imiglucerase to Velaglucerase Alfa Treatment in Patients With Type 1 Gaucher Disease.” American Journal of Hematology 90, no. 7: 592–597. 10.1002/ajh.24007. 25776130

[mgg370111-bib-0001] European Medicines Agency . 2010. “Guideline on the Investigation of Bioequivalence.” https://www.ema.europa.eu/en/investigation‐bioequivalence‐scientific‐guideline. 10.1111/j.1742-7843.2009.00518.x 20070293

[mgg370111-bib-0009] Grabowski, G. A. , G. Andria , A. Baldellou , et al. 2004. “Pediatric Non‐Neuronopathic Gaucher Disease: Presentation, Diagnosis and Assessment. Consensus Statements.” European Journal of Pediatrics 163, no. 2: 58–66. 10.1007/s00431-003-1362-0. 14677061

[mgg370111-bib-0010] Grabowski, G. A. , N. W. Barton , G. Pastores , et al. 1995. “Enzyme Therapy in Type 1 Gaucher Disease: Comparative Efficacy of Mannose‐Terminated Glucocerebrosidase From Natural and Recombinant Sources.” Annals of Internal Medicine 122, no. 1: 33–39. 10.7326/0003-4819-122-1-199501010-00005. 7985893

[mgg370111-bib-0034] Gupta, P. , and G. Pastores . 2018. “Pharmacological Treatment of Pediatric Gaucher Disease.” Expert Rev Clin Pharmacol 11, no. 12: 1183–1194. 10.1080/17512433.2018.1549486. 30444430

[mgg370111-bib-0011] Horowitz, M. , M. Pasmanik‐Chor , Z. Borochowitz , et al. 1998. “Prevalence of Glucocerebrosidase Mutations in the Israeli Ashkenazi Jewish Population.” Human Mutation 12, no. 4: 240–244. 9744474 10.1002/(SICI)1098-1004(1998)12:4<240::AID-HUMU4>3.0.CO;2-J

[mgg370111-bib-0012] Hwang, S. , H. Bae , J. H. Yoon , et al. 2024. “A 10‐Year Follow‐Up of High‐Dose Ambroxol Treatment Combined With Enzyme Replacement Therapy for Neuropathic Gaucher Disease.” American Journal of Hematology 99, no. 7: 1396–1399. 10.1002/ajh.27302. 38562044

[mgg370111-bib-0013] Kim, E. N. , H. S. Do , H. Jeong , et al. 2022. “Identification of a Novel Therapeutic Target Underlying Atypical Manifestation of Gaucher Disease.” Clinical and Translational Medicine 12, no. 5: e862. 10.1002/ctm2.862. 35593204 PMC9121313

[mgg370111-bib-0014] Kim, Y. M. , M. S. Yum , S. H. Heo , et al. 2020. “Pharmacologic Properties of High‐Dose Ambroxol in Four Patients With Gaucher Disease and Myoclonic Epilepsy.” Journal of Medical Genetics 57, no. 2: 124–131. 10.1136/jmedgenet-2019-106132. 31649052 PMC7029246

[mgg370111-bib-0015] Lee, B. H. , A. F. Abdalla , J. H. Choi , et al. 2017. “A Multicenter, Open‐Label, Phase III Study of Abcertin in Gaucher Disease.” Medicine (Baltimore) 96, no. 45: e8492. 10.1097/md.0000000000008492. 29137040 PMC5690733

[mgg370111-bib-0017] Mistry, P. K. , E. P. Wraight , and T. M. Cox . 1996. “Therapeutic Delivery of Proteins to Macrophages: Implications for Treatment of Gaucher's Disease.” Lancet 348, no. 9041: 1555–1559. 10.1016/s0140-6736(96)04451-0. 8950883

[mgg370111-bib-0016] Mistry, P. K. , M. D. Cappellini , E. Lukina , et al. 2011. “A Reappraisal of Gaucher Disease‐Diagnosis and Disease Management Algorithms.” American Journal of Hematology 86, no. 1: 110–115. 10.1002/ajh.21888. 21080341 PMC3058841

[mgg370111-bib-0018] Nabizadeh, A. , B. Amani , M. Kadivar , et al. 2018. “The Clinical Efficacy of Imiglucerase Versus Eliglustat in Patients With Gaucher's Disease Type 1: A Systematic Review.” Journal of Research in Pharmacy Practice 7, no. 4: 171–177. 10.4103/jrpp.JRPP_18_24. 30622983 PMC6298139

[mgg370111-bib-0019] Pastores, G. M. , S. P. Shankar , M. Petakov , et al. 2016. “Enzyme Replacement Therapy With Taliglucerase Alfa: 36‐Month Safety and Efficacy Results in Adult Patients With Gaucher Disease Previously Treated With Imiglucerase.” American Journal of Hematology 91, no. 7: 661–665. 10.1002/ajh.24399. 27102949 PMC5084808

[mgg370111-bib-0020] Ramaswami, U. , E. Mengel , A. Berrah , et al. 2021. “Throwing a Spotlight on Under‐Recognized Manifestations of Gaucher Disease: Pulmonary Involvement, Lymphadenopathy and Gaucheroma.” Molecular Genetics and Metabolism 133, no. 4: 335–344. 10.1016/j.ymgme.2021.06.009. 34229967

[mgg370111-bib-0021] Roshan Lal, T. , and E. Sidransky . 2017. “The Spectrum of Neurological Manifestations Associated With Gaucher Disease.” Diseases 5, no. 1: 10. 10.3390/diseases5010010. 28933363 PMC5456331

[mgg370111-bib-0022] Serratrice, C. , S. Carballo , J. Serratrice , and J. Stirnemann . 2016. “Imiglucerase in the Management of Gaucher Disease Type 1: An Evidence‐Based Review of Its Place in Therapy.” Core Evidence 11: 37–47. 10.2147/ce.S93717. 27790078 PMC5072572

[mgg370111-bib-0023] Shemesh, E. , L. Deroma , B. Bembi , et al. 2015. “Enzyme Replacement and Substrate Reduction Therapy for Gaucher Disease.” Cochrane Database of Systematic Reviews 2015, no. 3: Cd010324. 10.1002/14651858.CD010324. 25812601 PMC8923052

[mgg370111-bib-0024] Smith, L. , W. Rhead , J. Charrow , et al. 2016. “Long‐Term Velaglucerase Alfa Treatment in Children With Gaucher Disease Type 1 naïve to Enzyme Replacement Therapy or Previously Treated With Imiglucerase.” Molecular Genetics and Metabolism 117, no. 2: 164–171. 10.1016/j.ymgme.2015.05.012. 26043810

[mgg370111-bib-0025] Starzyk, K. , S. Richards , J. Yee , S. E. Smith , and W. Kingma . 2007. “The Long‐Term International Safety Experience of Imiglucerase Therapy for Gaucher Disease.” Molecular Genetics and Metabolism 90, no. 2: 157–163. 10.1016/j.ymgme.2006.09.003. 17079176

[mgg370111-bib-0026] Stirnemann, J. , N. Belmatoug , F. Camou , et al. 2017. “A Review of Gaucher Disease Pathophysiology, Clinical Presentation and Treatments.” International Journal of Molecular Sciences 18, no. 2: 441. 10.3390/ijms18020441. 28218669 PMC5343975

[mgg370111-bib-0027] Tajima, A. , T. Yokoi , M. Ariga , et al. 2009. “Clinical and Genetic Study of Japanese Patients With Type 3 Gaucher Disease.” Molecular Genetics and Metabolism 97, no. 4: 272–277. 10.1016/j.ymgme.2009.05.001. 19481486

[mgg370111-bib-0028] Weinreb, N. J. 2008. “Imiglucerase and Its Use for the Treatment of Gaucher's Disease.” Expert Opinion on Pharmacotherapy 9, no. 11: 1987–2000. 10.1517/14656566.9.11.1987. 18627336

[mgg370111-bib-0029] Weinreb, N. J. , J. Goldblatt , J. Villalobos , et al. 2013. “Long‐Term Clinical Outcomes in Type 1 Gaucher Disease Following 10 Years of Imiglucerase Treatment.” Journal of Inherited Metabolic Disease 36, no. 3: 543–553. 10.1007/s10545-012-9528-4. 22976765 PMC3648688

[mgg370111-bib-0033] Zimran, A. , D. Elstein , E. Levy‐Lahad , et al. 1995. “Replacement Therapy With Imiglucerase for Type 1 Gaucher's Disease.” Lancet 345, no. 8963: 1479–1480. 10.1016/s0140-6736(95)91038-7. 7769903

[mgg370111-bib-0032] Zimran, A. , E. Brill‐Almon , R. Chertkoff , et al. 2011. “Pivotal Trial With Plant Cell‐Expressed Recombinant Glucocerebrosidase, Taliglucerase Alfa, a Novel Enzyme Replacement Therapy for Gaucher Disease.” Blood 118, no. 22: 5767–5773. 10.1182/blood-2011-07-366955. 21900191

[mgg370111-bib-0030] Zimran, A. , G. Altarescu , M. Philips , et al. 2010. “Phase 1/2 and Extension Study of Velaglucerase Alfa Replacement Therapy in Adults With Type 1 Gaucher Disease: 48‐Month Experience.” Blood 115, no. 23: 4651–4656. 10.1182/blood-2010-02-268649. 20299511

[mgg370111-bib-0031] Zimran, A. , N. Belmatoug , B. Bembi , et al. 2018. “Demographics and Patient Characteristics of 1209 Patients With Gaucher Disease: Descriptive Analysis From the Gaucher Outcome Survey (GOS).” American Journal of Hematology 93, no. 2: 205–212. 10.1002/ajh.24957. 29090476 PMC5814927

